# BIRICODAR (VX-710; Incel™): an effective chemosensitizer in neuroblastoma

**DOI:** 10.1038/sj.bjc.6990485

**Published:** 1999-06

**Authors:** T Yanagisawa, A Newman, H Coley, J Renshaw, C R Pinkerton, K Pritchard-Jones

**Affiliations:** Section of Paediatrics, The Institute of Cancer Research/Royal Marsden NHS Trust, Sutton, Surrey, SM2 5NG, UK; CRC Centre for Cancer Therapeutics, The Institute of Cancer Research, Sutton, Surrey, SM2 5NG, UK

## Abstract

Clinical studies have suggested that both MDR1 and MRP may play a significant role in the chemosensitivity and outcome of neuroblastoma. To clarify the nature of multidrug resistance (MDR) in this tumour a series of six neuroblastoma cell lines have been characterized with regard to P-glycoprotein, MRP and LRP expression using immunocytochemistry and expression of MDR1, MRP, LRP and topoisomerase II genes using reverse transcription polymerase chain reaction (RT-PCR). By RT-PCR, all lines expressed MRP, five expressed LRP and four expressed MDR1, but protein levels of each of these were variable. Chemosensitization to a range of MDR-associated drugs (vincristine, doxorubicin, etoposide, taxotere, topotecan) and non-MDR-associated drugs (cisplatin, melphalan) by three modulating agents, cyclosporin A, PSC 833 and the novel Biricodar (VX-710; Incel™), was evaluated using a colourimetric cytotoxicity assay (MTS). Alteration of daunorubicin efflux by these agents was evaluated using FACS analysis. Clonogenic assay was used to study the influence of these chemosensitizers on vincristine cytotoxicity. Marked sensitization to vincristine was observed in MDR1-positive lines, and a similar but less consistent effect was seen with taxotere, doxorubicin and etoposide. With MRP-positive, MDR-negative lines, only VX-710 caused consistent sensitization. These data confirm MDR1 and MRP expression as contributory factors in chemoresistance in neuroblastoma and indicate that VX-710 may be a useful modulator of both mechanisms and worthy of clinical evaluation in this tumour. © 1999 Cancer Research Campaign


*British Journal of Cancer* (1999) **80**(8), 1190–1196
© 1999 Cancer Research Campaign
Article no. bjoc.1999.0485

### BIRICODAR (VX-710; Incel™): an effective chemosensitizer in neuroblastoma

**T Yanagisawa[1], A Newman[1], H Coley[2], J Renshaw[1], CR Pinkerton[1] and K Pritchard-Jones[1]**

[1]Section of Paediatrics, The Institute of Cancer Research/Royal Marsden NHS Trust, Cotswold Road, Sutton, Surrey SM2 5NG, UK; [2]CRC Centre for Cancer Therapeutics, The Institute of Cancer Research, Cotswold Road, Sutton, Surrey SM2 5NG, UK

**Summary** Clinical studies have suggested that both MDR1 and MRP may play a significant role in the chemosensitivity and outcome of neuroblastoma. To clarify the nature of multidrug resistance (MDR) in this tumour a series of six neuroblastoma cell lines have been characterized with regard to P-glycoprotein, MRP and LRP expression using immunocytochemistry and expression of MDR1, MRP, LRP and topoisomerase II genes using reverse transcription polymerase chain reaction (RT-PCR). By RT-PCR, all lines expressed MRP, five expressed LRP and four expressed MDR1, but protein levels of each of these were variable. Chemosensitization to a range of MDR-associated drugs (vincristine, doxorubicin, etoposide, taxotere, topotecan) and non-MDR-associated drugs (cisplatin, melphalan) by three modulating agents, cyclosporin A, PSC 833 and the novel Biricodar (VX-710; Incel™), was evaluated using a colourimetric cytotoxicity assay (MTS). Alteration of daunorubicin efflux by these agents was evaluated using FACS analysis. Clonogenic assay was used to study the influence of these chemosensitizers on vincristine cytotoxicity. Marked sensitization to vincristine was observed in MDR1-positive lines, and a similar but less consistent effect was seen with taxotere, doxorubicin and etoposide. With MRP-positive, MDR-negative lines, only VX-710 caused consistent sensitization. These data confirm MDR1 and MRP expression as contributory factors in chemoresistance in neuroblastoma and indicate that VX-710 may be a useful modulator of both mechanisms and worthy of clinical evaluation in this tumour.

Although half of all children with neuroblastoma will be cured of their disease, there are still subgroups in whom the outlook remains poor. These include children with metastatic disease, particularly affecting bone, or those with regional disease in whom the tumour shows N-MYC amplification or chromosome 1p deletion. In these patients, despite intensive multiagent chemotherapy often followed by high dose chemotherapy with stem cell rescue, less than one-quarter will be long-term survivors. Since the introduction of cisplatin–etoposide combinations there have been few new chemotherapy agents which have proven to be active in neuroblastoma. Alternative strategies, such as dose escalation or high dose intensity regimens, may have improved remission rates but have had only modest impact on cure rates. There is therefore an urgent need for new approaches in this disease.

As in other childhood tumours, drug resistance in neuroblastoma is multifactorial, but there is evidence that multidrug resistance (MDR) involving P-glycoprotein (P-gp), the product of the MDR1 gene or MDR-related protein, the product of the MRP gene may influence both the biological behaviour of these tumours and response to chemotherapy (Bourhis et al, 1989; Chan et al, 1991; Bordow et al, 1994; Norris et al, 1996). Resistance to several of the most important drugs used in children's cancer, for example anthracyclines, vincristine, etoposide and actinomycin, has been shown to involve these mechanisms.

Modulation of P-gp function has been demonstrated in a range of tumour cell lines using a variety of compounds. The most extensively evaluated in clinical practice are verapamil and cyclosporin A (Cairo et al, 1989; Lum et al, 1993; Cowie et al, 1994, 1995; Chan et al, 1995). The cyclosporin analogue PSC 833 has been introduced more recently (Helson et al, 1994; Boote et al, 1996) and is currently undergoing a dose-finding study in children. The major drawback of MDR modulation strategies thus far has been the unacceptable level of toxicity of the modulating agent. In the case of verapamil, it has proven difficult to achieve blood drug levels in patients comparable to those shown to be effective in vitro due to cardiac toxicity. Similarly, with high dose cyclosporin, dose-limiting nephrotoxicity and hepatic toxicity have compounded the extramedullary toxicity of the cytotoxic chemotherapy. There is, therefore, a need for alternative reversal agents with a lesser toxicity to normal tissue.

VX-710 is a non-macrocyclic pipecolinate derivative which binds the FK506 receptor protein. VX-710 has been shown to restore sensitivity in a range of multidrug-resistant cells, including myeloma, melanoma, carcinoma and leukaemia (Germann et al, 1997*a*). The drug inhibits the efflux activity of P-gp and stimulates vanadate-sensitive P-gp ATPase activity, suggesting a direct high affinity interaction between VX-710 and P-gp (Germann et al, 1997*a*). Of particular importance are results from Phase I clinical studies in adults which have shown this agent to have an encouraging toxicity profile with doses up to 120 mg $m^{-2}$ per hour as a 24 to 96-h infusion. Minimal adverse reactions. These included headaches, mild to moderate nausea and mild reversible hypotension. At the highest dose there was mild hyperbilirubinaemia (Rowinsky et al, 1998). Steady-state concentrations of VX-710 (≥8 μM) were 2–3 fold higher than level effective in vitro as an MDR modulator. Moreover, hepatic retention of sestamibi increased up to eightfold during VX-710 administration, suggesting inhibition of P-gp in the liver (Peck et al, 1995).

In the present study, a range of neuroblastoma cell lines have been characterized with regard to drug resistance phenotype and the chemosensitization to several cytotoxic agents using PSC 833, cyclosporin and VX-710. Three methods were used: microtitre

*Received 12 January 1998*
*Revised 13 January 1999*
*Accepted 18 January 1999*

*Correspondence to:* K Pritchard-Jones

**Table 1** Clinical data on origins of the neuroblastoma cell lines

| Cell line | Patient age (months) | Tissue source | Prior therapy | Response to therapy |
|---|---|---|---|---|
| SKNBE | 24 | Bone marrow | RT, VCR, CTX, ADR, ACT | None |
| SKNSH | 48 | Bone marrow | RT, VCR, CTX, DNR, FMdU, ADR | None |
| HX-142 | 19 | Primary tumour | VCR, CDDP, VM-26, CTX | PR |
| HX-138 | 72 | Primary tumour | RT, VCR, CDDP, ETOP, CTX | PR |
| NB-1 | 33 | Cervical lymph node | RT, ACT, CTX, VCR | PR |
| IMR32 | 13 | Primary tumour | None | |

RT = radiotherapy (specimens were not obtained from within the radiation port); VCR = vincristine; CTX = cyclophosphamide; ADR = doxorubicin; ACT = dactinomycin; DNR = daunomycin; FMdU = trifluoromethyl-2′-deoxyuridine; CDDP = cisplatin; VM-26 = teniposide; ETOP = etoposide; DTIC = dacarbazine; PR = partial response.

tetrazolium–formazan assay (MTS), clonogenic assay and daunorubicin efflux with FACS.

### METHODS

### Cell lines and culture conditions

The derivation and characterization of the six established human neuroblastoma cell lines used in this study are outlined in Table 1. SKNBE, IMR-32, SKNSH and SKNBE cells were obtained from the American Tissue Culture Collection (Rockville, MD, USA). NB-1 cells were obtained from the Japanese Cancer Research Resources Bank (Tokyo, Japan). Hx-138 and Hx-142 were provided by Professor G Steele (Radiotherapy Unit, Institute of Cancer Research, Sutton, UK) (Tumilowicz et al, 1970; Biedler et al, 1973; Inui, 1974; Biedler and Spengler, 1976; Deacon, 1986; Hills et al, 1989; Feller et al, 1995). The doxorubicin-resistant, non-small-cell lung cancer cell line, 2R120, was used as a positive control for MRP expression and provided a negative control for P-gp. The sensitive human ovarian carcinoma line CH-1 and the doxorubicin-resistant derivative line CH-1R were used as negative and positive controls for MDR-1 expression. Cell lines were maintained as monolayers and incubated at 37°C in a humidified atmosphere with 5% carbon dioxide in culture media supplemented with 10% heat-inactivated fetal bovine serum (FBS; GibcoBRL, UK), 1 ml 100 $ml^{-1}$ Glutamax (Gibco), 100 U $ml^{-1}$ of penicillin and 100 μg $ml^{-1}$ streptomycin. RPMI-1640 medium (Gibco) was used for SKNBE, SKNSH, IMR-32 and NB-1, Ham's F12 (Gibco) for HX-138 and HX-142, and DMEM (Gibco) for all control cell lines. Maintenance doses of doxorubicin were used to retain the MDR phenotype of the drug-resistant cell lines until at least 48 h before the start of experiments. All cell lines were routinely screened for mycoplasma by PCR assay (Mycoplasma Primer Set, Stratagene, Cambridge, UK).

#### *MDR characterization by immunocytochemistry*

The consensus recommendations of the multicentre workshop were applied, where possible, in this study (Beck et al, 1996). Two antibodies raised against spatially distinct segments of each protein were used for the immunocytochemical detection and semi-quantitative measurement of MRP and P-gp. Control reactions used appropriately matched isotypes or no primary antibody. Cytospin preparations of 50 000 cells were fixed in acetone at –20°C for 5 min, air-dried, preincubated in 10% human AB serum in phosphate-buttered saline (PBS) A, followed by incubation with the primary antibody for 1 h. For detection of P-gp, the mouse monoclonal antibodies, MRK 16 (TCS Biologicals Ltd, Boltolph Claydon, UK), 1:25 dilution, JSB –1, 1:10 and for MRP the antibodies MRPr1, raised in rat (Sigma Chemicals, Poole, UK), dilution 1:50 and MRPm6 (kindly provided by Dr Rik Scheper, Free University, Amsterdam, The Netherlands) raised in mouse at 1:25 were used. The second antibody, rabbit anti-mouse or anti-rat (Dako, Glastrup, Denmark) was used at 1:50 dilution for 30 min. A standard APAAP technique (DAKO) was used for colourimetric development with levamisole (Sigma) to block activity of endogenous alkaline phosphatase. All incubations were at room temperature. Slides were counterstained with Mayer's haematoxylin (Sigma, 2 min). Cells were graded as negative when no staining was apparent and from (+) to (++++), according to the intensity of staining (Beck et al, 1996). Positive control cell lines scored (+++) or (++++) for MRP and P-gp. Reproducibility of the results was confirmed in each case by two repeat experiments.

#### *Reverse transcriptase polymerase chain reaction*

***Oligonucleotides*** All sequences of polymerase chain reaction (PCR) primers were as previously reported. Reverse transcription (RT)-PCR analysis of MDR1 and MRP gene expression was performed using two non-overlapping primer pairs, which gave concordant results for each gene in all cell lines (MDR-1: 401-bp product (Futscher et al, 1993); 157-bp product (Bordow et al, 1994); MRP: 565-bp product (Beketic-Oreskovic et al, 1995); 140-bp product (Bordow et al, 1994)). Other primer sets were: topoisomerase II α (topo II α), 322-bp product (Beck et al, 1995), topoisomerase II β (topo II β), 304-bp (Beck et al, 1995), lung-related resistance protein (LRP), 405-bp (Stein et al, 1997), glyceraldehyde-3-phosphate dehydrogenase (GAPDH), 358-bp and $\beta_2$ microglobulin ($\beta_2$M), 120-bp (Bordow et al, 1994). All primer sets spanned an intron to control against amplification of genomic DNA sequences. Two internal controls (GAPDH and $\beta_2$M) were used to assess the quality of template in each reaction. All primers were purchased from Oswel DNA Service Lab (Southampton, UK).

Total RNA (1 μg) was reverse transcribed in a 20 μl volume using Superscript II (GibcoBRL) and random hexanucleotides (Pharmacia), according to the manufacturers' instructions. PCR was performed on 50 ng of cDNA in a final volume of 50 μl containing 25 pmol of each primer, 0.4 mM dNTPs (Pharmacia), 20 mM Tris–HCl, 50 mM potassium chloride, 1.8 mM magnesium chloride and 2.5 U $\mu l^{-1}$ of *Taq* DNA polymerase (Gibco BRL). An initial denaturation for 4 min at 94°C was followed by 27–35 cycles of 45 s at 94°C, 45 s at 55°C, 90 s at 72°C in an Omnigene

© 1999 Cancer Research Campaign

**Table 2** Expression of MDR-related genes in neuroblastoma and control cell lines

| Cell line | Immunocytochemistry staining | | | | | RT-PCR | | |
|---|---|---|---|---|---|---|---|---|
| | MDR-1 | | MRP | | LRP | MDR-1 | MRP | LRP |
| | MRK16 | JSB-1 | MRP-6 m | MRPr-1 | LRP-56 | | | |
| SKNBE | (+++) | (+++) | (+) | (+++) | (–) | + | + | + |
| SKNSH | (+++) | (+++) | (+) | (+++) | (–) | + | + | + |
| HX142 | (+) | (+) | (++) | (+++) | (–) | + | + | – |
| HX138 | (+) | (+) | (+) | (++++) | (+++) | + | + | + |
| NB1 | (++) | (++) | (+) | (+) | (–) | – | + | + |
| IMR 32 | (–) | (–) | (–) | (–) | (–) | – | + | + |
| CH-1P | (–) | (–) | (–) | (–) | (–) | – | + | + |
| CH-1R | (+++) | (++++) | (–) | (–) | (–) | + | + | + |
| 2R120 | (–) | (–) | (+++) | (+++) | (+++) | – | + | + |

Immunocytochemistry – intensity of staining recorded as: negative (–), low (+), intermediate (++), high (+++), ultra high (++++). RT-PCR – for MDR-1 and MRP two different primer pairs were used. See Methods.

thermal cycler (Hybaid, UK). PCR products (20 μl) were visualized by gel electrophoresis (2% agarose or 12% polyacrylamide) and ethidium bromide staining. Each sample was assayed at least twice and scored as positive if the correct sized PCR product was detected in two or more experiments with appropriate negative controls (no RNA, no DNA).

### *Chemicals*

Drug sources were as follows: vincristine (Farmitalia Carlo Erba Ltd, Herts, UK), doxorubicin (Eli Lilly, Basingstoke, UK), melphalan and etoposide (Sigma, UK), cisplatin (Johnson Matthey, Reading, UK), taxotere (Rhone-Poulenc, France), topotecan (SmithKline Beecham Pharmaceuticals, Herts, UK), cyclosporin A and PSC 833 (Novartis Pharmaceuticals, Camberley, UK), VX-710 (Vertex Pharmaceuticals Inc., Cambridge, MA, USA). Stock solutions were prepared in saline or water with the exception of: (1) melphalan, which was solubilized in a minimum quantity of 4.5% ethanolic hydrochloric acid and then diluted at least 50-fold in medium before addition to cultures; (2) etoposide and PSC 833, which were solubilized in absolute alcohol and similarly diluted; (3) VX-710 was diluted in the diluent DMEM. Stock solutions were diluted with tissue culture media just before use. The levels of the diluted solvents alone showed no toxic effect on cells (data not shown).

### *Functional analysis of P-gp and MRP*

Cells in single cell suspension (0.5–1.0 × $10^6$ in 1 ml aliquots) were equilibrated for 30–60 min at 37°C in the absence/presence of modulating agents (PSC 833 500 nM, VX-710 5 μM) in Dulbecco's phenol-red free medium with 10% FCS. Daunorubicin (Sigma Chemicals), at a concentration of 5 μM was then added, with a further 60 min incubation. Following the fluorescent probe accumulation phase, cells were pelleted by centrifugation at 2000 ***g***, 4°C, and resuspended in fresh medium, again in the absence or presence of the appropriate modulating agent. Cells were allowed to efflux for a further 60 min at 37°C and were then pelleted as before. Following resuspension in drug-free medium, cells were stored on ice prior to analysis. Throughout the incubation periods, cells were gently agitated at regular intervals to prevent excessive clumping. A control untreated cell suspension was run at the beginning of each analysis, to allow elimination of clumped cells and debris by gating out. Fluorescence was analysed for 10 000 events using a Coulter Epics ESP Elite flow cytometer.

### *Cytotoxicity assays*

The drug sensitivity and the potencies of the modulators were determined by the MTS cytotoxicity assay (Promega) for all cell lines and clonogenic assay in SKNBE (Goodwin et al, 1995). Cells were seeded at 2500–5000 cells per well to allow exponential growth over not more than four doublings during the incubation period. Plates were preincubated for 24 h, then cytotoxic drugs with or without modulator were added to a final volume of 200 μl. Modulators were used at a concentration that produced less than 10% cell death (PSC833 and cyclosporin A were used at 0.5 μM for all lines; VX710 was used at 5 μM for all lines except HX138 (2.0 μM) and NB-1 (1.0 μM)). Control wells contained medium only or cells alone. All experimental points were set up in quadruplicate wells and all experiments were repeated at least three times. The MTS assay was performed after 72–156 h, depending on the doubling time, to obtain an $IC_{50}$ for each drug. The resistance modifying factor or sensitization ratio was calculated as ($IC_{50}$ with drug alone)/($IC_{50}$ drug + modulator).

Clonogenic assays were performed using a modification of Pike and Robinson's double layer agar technique (Pike and Robinson, 1970). Five hundred neuroblastoma cells were plated in the presence of 0.0001, 0.001, 0.01 and 1.0 μM vincristine alone or with modulator (0.5 μM cyclosporin A or PSC 833, 5.0 μM VX-710). Cell suspensions were prepared by trypsinization of monolayers and gently passing through a 21-gauge needle. The effectiveness of this procedure to achieve a suspension of single cells was checked microscopically. Each drug concentration and modulator combination was set up in triplicate. Plates were incubated for 14 days. Aggregates of more than 50 cells were scored as colonies. Survival was expressed as a percentage of control colony counts.

The nonparametric, two-sided Mann–Whitney rank sum test was used to analyse the MTS assay data, comparing median sensitization ratios from at least three independent experiments for each data point. Statistical significance was established on the basis of 95% confidence intervals.

### RESULTS

### MDR expression

Expression of three genes implicated in multidrug resistance (MDR-1, MRP, LRP) was assayed at both the mRNA and protein level in six neuroblastoma and three control cell lines (Table 2). Topoisomerase II α or β were assayed solely by RT-PCR.

© 1999 Cancer Research Campaign

**Table 3** Sensitization of neuroblastoma cell lines to cytotoxic agents by cyclosporin A, PSC 833 and VX710

| Cell line | Vincristine | | | Taxotere | | | Doxorubicin | | | Etoposide | | |
|---|---|---|---|---|---|---|---|---|---|---|---|---|
| | CsA | PSC833 | VX710 | CsA | PSC833 | VX710 | CsA | PSC833 | VX710 | CsA | PSC833 | VX710 |
| SKNBE | 26 | 33 | 61 | 8.3 | 8.3 | 14 | 2.5 | 3.1 | 2.8 | 2.4 | 2.2 | 2.8 |
| SKNSH | 6.8 | 7.4 | 32 | 4.7 | 5.8 | 7.6 | 2.4 | 2.4 | 3.1 | 1.3 | 1.5 | 1.4 |
| Hx142 | 6.8 | 5.3 | 12 | 3.3 | 3.4 | 3.3 | 1.8 | 1.5 | 2.4 | 3.3 | 2.8 | 3.5 |
| Hx138 | 5.2 | 4.6 | 6.7 | 5.5 | 4.1 | 2.4 | 2.4 | 2.0 | 3.5 | 2.0 | 3.5 | 1.1 |
| NB 1 | 1.2 | 1.0 | 1.6 | 1.5 | 0.9 | 1.2 | 1.1 | 0.8 | 1.8 | 1.0 | 1.4 | 1.0 |
| IMR-32 | 1.0 | 0.7 | 1.5 | 1.2 | 1.0 | 1.0 | 1.0 | 0.9 | 0.9 | 1.0 | 1.3 | 1.4 |

Data shown are median sensitization ratios (SR) from at least three independent experiments. SR = $IC_{50}$ with drug/$IC_{50}$ drug + modulator. $IC_{50}$ values were calculated from dose–response curves of MTS cytotoxicity assays. Modulators (cyclosporin A (CsA), PSC 833 (PSC) and VX710 (VX)) were used at concentrations <$IC_{10}$ for each cell line.

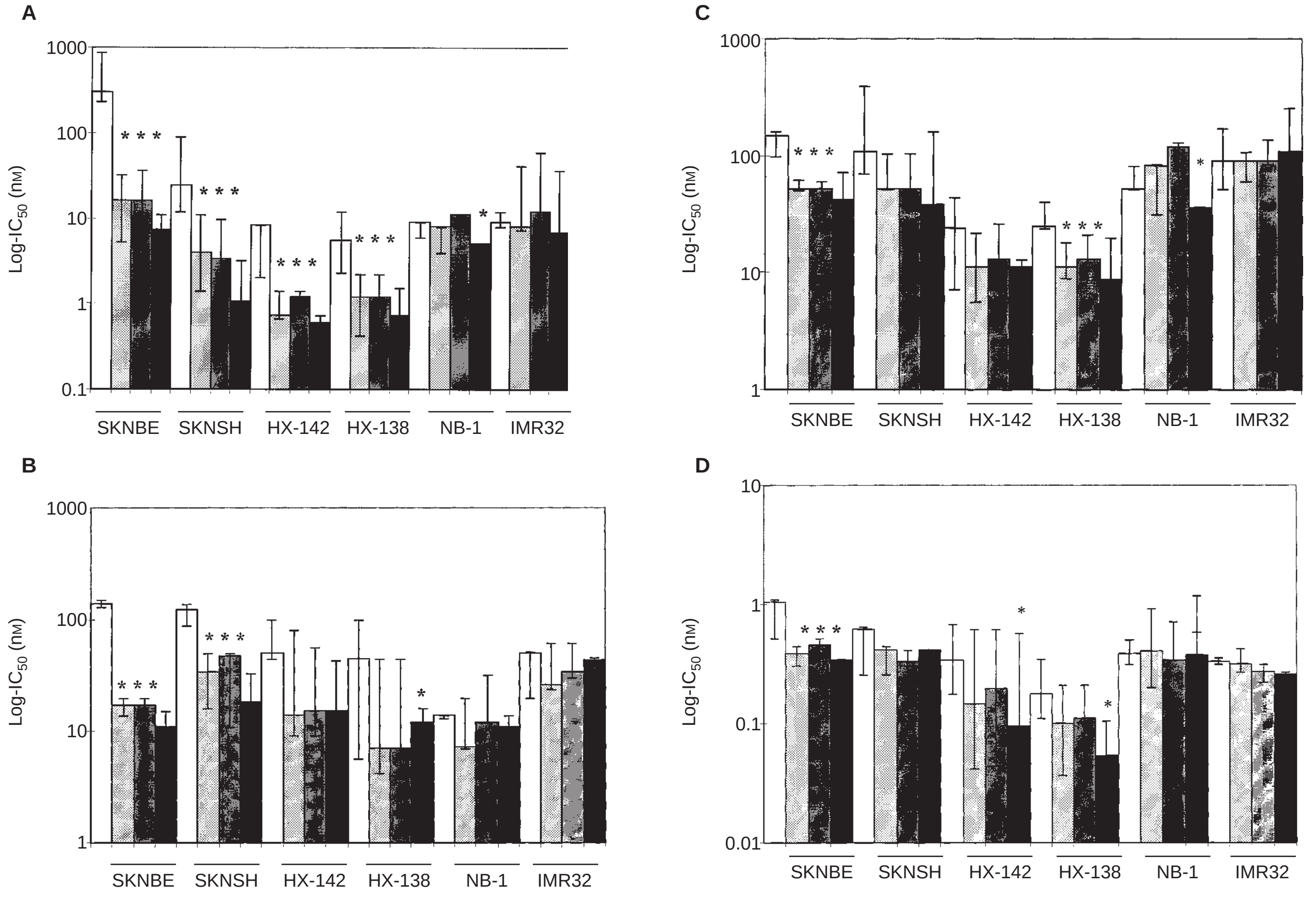

**Figure 1** Effects of modulators on cytotoxicity of (**A**) vincristine, (**B**) taxotere, (**C**) doxorubicin and (**D**) etoposide in neuroblastoma cell lines. The results are presented as the median with the range of $IC_{50}$ values obtained from at least three independent experiments. Median $IC_{50}$ values in the presence of modulator which are statistically significant (non-parametric Mann–Whitney rank sum test, $P < 0.05$) from those for the drug alone are asterisked. □, Drug; alone, ▨, +CsA; ■, +PSC833; ■, +Vx710

P-gp expression was detected in 5/6 neuroblastoma cell lines and MDR-1 mRNA by RT-PCR in 4/6. NB1 cells gave a discordant result that was probably due to artefactual binding or cross-reactivity in immunocytochemistry. RT-PCR with two non-overlapping primer sets was consistently negative and P-gp could not be detected by either Western or flow cytometric analysis in this cell line (data not shown). In functional assays, NB1 behaved similarly to IMR32, which was MDR-1/P-gp negative by all methods.

Five lines (all except IMR32) expressed MRP detectable by immunohistochemistry. However, all expressed MRP mRNA detectable by RT-PCR, presumably reflecting the greater sensitivity of the latter technique.

All neuroblastoma lines except one (HX-142) showed expression of LRP by RT-PCR but mainly at low levels, as only one (HX-138) had detectable protein by immunohistochemistry. No cell line showed loss of topoisomerase II α or β expression by RT-PCR.

*© 1999 Cancer Research Campaign*

**Table 4** Functional assay of effect of modulators on daunorubicin efflux

| Cell line | Efflux ratio | |
|---|---|---|
| | PSC 833 | VX710 |
| SKNBE | 1.5 | 1.1 |
| | (0.21) | (0.14) |
| SKNSH | 1.2 | 1.3 |
| | (0.19) | (0.19) |
| Hx142 | 2.4 | 2.2 |
| Hx138 | 1.3 | 1.2 |
| NB1 | 1.4 | 1.2 |
| | (0.16) | (0.06) |
| IMR32 | 1.2 | 1.3 |
| | (0.10) | (0.16) |
| CH-1 Pa | 1.0 | 1.0 |
| CH-1 Re | 6.2 | 6.0 |
| | (0.58) | (0.79) |
| 2R120 | 1.6 | 1.7 |
| | (0.26) | (0.11) |

Efflux ratio = daunorubicin fluorescence in the presence of modulator/daunorubicin fluorescence alone. Data points are either the mean of three or more experiments with standard deviation in parentheses or the mean of two experiments.

### Reversibility of drug resistance by MDR modulators

The ability of the three modulators, cyclosporin A, PSC 833 and VX-710, to overcome resistance to a panel of drugs, was examined in all cell lines by an MTS assay. The drug panel included five drugs derived from natural products which are expected to be transported by P-gp (vincristine, doxorubicin, etoposide, taxotere and topotecan) and two drugs (melphalan and cisplatin) which are not. All of these drugs, with the exception of taxotere and topotecan, are commonly used in the treatment of neuroblastoma. In the MTS assay, modulation values were expressed as a sensitization ratio (SR): ($IC_{50}$ drug alone)/($IC_{50}$ drug + modulator) (Table 3). Examples of histograms of median $IC_{50}$ are illustrated for the four drugs for which statistically significant modulation was detected in the neuroblastoma cell lines (Figure 1 A–D).

The degree of modulation was most marked with vincristine (up to 61-fold) and for VX710 often exceeded the level seen in the MDR-1 positive control cell line, CH-1R. No modulation of $IC_{50}$ was seen in the sensitive control cell line, CH1-P. Modulation of sensitivity to etoposide, doxorubicin and taxotere was variable and generally much less marked (up to 14-fold) than with vincristine. Of interest, no modulation of topotecan sensitivity was seen, although previous studies have demonstrated that topotecan can be transported by P-gp (Hendriks et al, 1992). No modulation was seen in any line with melphalan or cisplatin (Table 3 and data not shown).

The relative efficacy of the three modulators in reversing drug resistance was variable. VX-710 was generally the most effective modulator in the neuroblastoma cell lines, whereas PSC833 gave superior modulation in the two positive control cell lines. Both were generally superior to cyclosporin A.

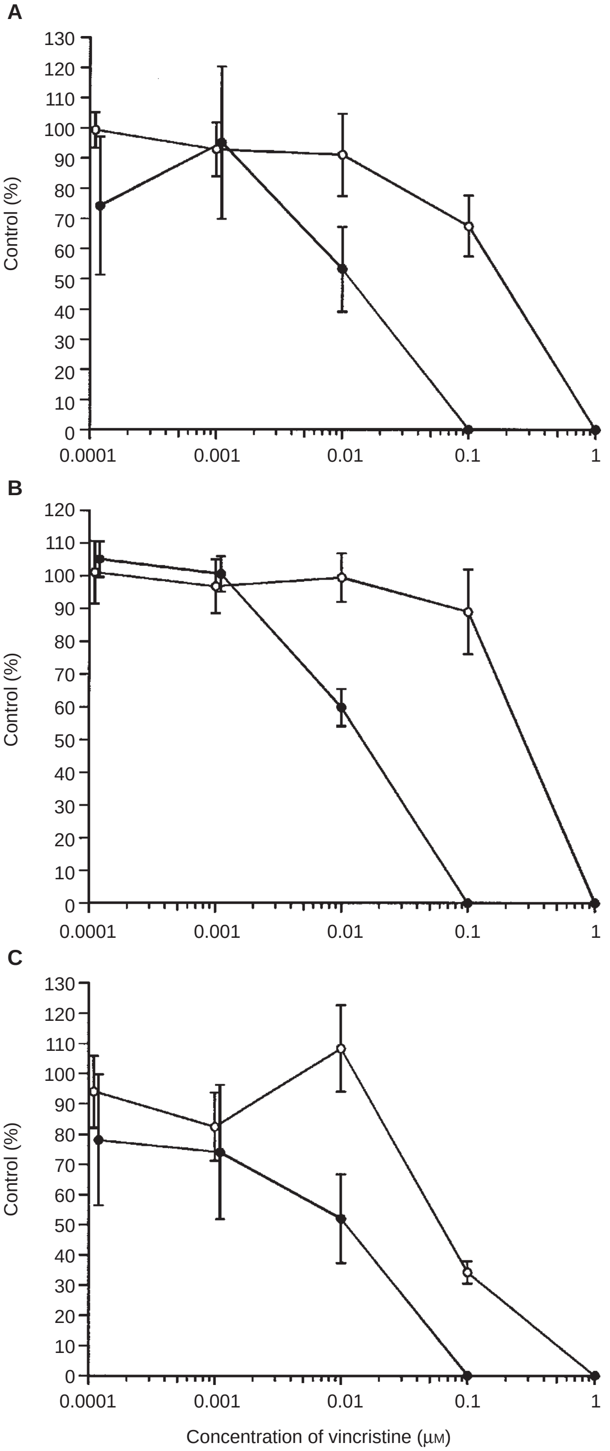

**Figure 2** Survival of clonogenic SKNBE neuroblasts exposed to vincristine alone or in combination with modulators. –○– without modulator, –●– with modulator (A = 0.5 μm CSA, B = 0.5 μm, PSC 833, C = 5 μm VX710)

### Correlation of modulation of drug resistance with P-gp and MRP expression

No cell line was completely ‘clean’ in having only one possible mechanism of drug resistance. Such multiplicity of mechanisms is probably common in vivo as well as in vitro and makes simple correlations impossible. All three modulators sensitized both the P-gp- and MRP-positive control cell lines to doxorubicin. The extremely high level of vincristine resistance in the MRP-positive control line 2R120 was not influenced by the modulators, implying additional resistance mechanisms for this drug in this cell line. VX710 gave the greatest number of significant modulations

 © 1999 Cancer Research Campaign across the drug panel. In the four neuroblastoma cell lines with both P-gp and MRP expression, VX-710 was the only modulator to significantly sensitize two lines to etoposide and one line to taxotere. VX710 was the only modulator to show an effect in line NB-1, whose drug resistance was ascribed to MRP rather than P-gp (SR = 1.6 for vincristine and 1.8 for doxorubicin).

### Clonogenic and functional assays

A subset of modulators and cell lines was further examined by two complementary assays: a clonogenic assay to assess modulation of cytotoxicity on clonogenic cells and a functional assay of drug efflux. The clonogenic assay was only performed for SKNBE exposed to vincristine in combination with each of the three modulators (Figure 2). These results show that all three modulators enhance the killing of clonogenic cells in addition to the overall enhancement of cytotoxicity demonstrated by the MTS assays. Using the functional assay, retention of daunorubicin was clearly enhanced in the CH-1R Pgp-positive control line by both PSC833 and VX-710 (Table 4). This assay showed a more modest degree of modulation in the neuroblastoma cell lines and the MRP-positive control for both VX-710 and PSC 833.

### DISCUSSION

This study characterized the MDR phenotype of a range of neuroblastoma cell lines and documented the activity of VX-710 as a drug resistance modulator compared to cyclosporin A and PSC 833. The secondary objective was to determine to what extent any sensitization was linked with known mechanisms of MDR.

As expected from the discussions of the multicentre workshop (Beck et al, 1996) there was some discordance between the characterization of cell lines using different methods. In general, there was a high level of concordance between RT-PCR and immunocytochemistry for both MDR1 and MRP. The staining intensity for both MDR1 and MRP varied depending on the antibody used (Table 2). Discordance occurred only in the case of NB-1 where gene expression was not apparent on RT-PCR despite apparent immunoreactivity. The lack of modulation with cyclosporin A and PSC 833 together with the observation that no significant P-gp levels were detected by either Western of FACS analysis implies that the immunocytochemical staining was a false positive. Functionally significant MRP expression appeared to correlate with positivity in immunocytochemistry rather than RT-PCR. IMR32 and CH-1P were only MRP-positive by RT-PCR and showed no evidence of modulation. All neuroblastoma cell lines expressed the LRP gene but most did not have detectable protein. In no cell line was there evidence of complete absence of topoisomerase II expression by RT-PCR, although a reduction in level of expression would not be detected by this method.

Cytotoxicity and modulation was evaluated using three independent methods, MTS, clonogenic assay and drug efflux modulation. The commonly used MTS or MTT assays have the limitation of assessing overall cell kill, not necessarily cells capable of repopulation. In this study there was close correlation between the modulation observed with all three modulators as evaluated by either MTS or clonogenic assay. Efflux assay using either daunorubicin or rhodamine may shed light on the mechanism by which VX-710 influences cytotoxicity. It is clear, however, that changes in drug efflux are not necessarily translated into cell kill. The degree of sensitization to anthracyclines using VX-710 or PSC 833 were more marked with MTS assay than might have been predicted with functional assay, particularly in SKNBE and HX138. A close correlation between cytotoxicity as measured by MTS assay and drug efflux was apparent for the P-gp control cell lines. The sensitive parental line, CH1-P, showed no evidence of modulation whereas in the resistant line, CH-1R, marked enhancement of drug retention was reflected in high levels of cytotoxicity to both vincristine and doxorubicin. In clinical practice, the functional assay is generally the method of choice for evaluating leukaemia, where fresh tumour cells can be readily evaluated. For solid tumours this is difficult and the MTS assay may be more practical.

In the present study the highest dose used for the VX-710 studies was 5 μM. This is a concentration which is readily achieved in clinical studies (Rowinsky et al, 1998). Even lower concentrations may be effective as dose–response studies (data not shown) failed to show any significant difference in the modulation of SKNBE with vincristine over a VX-710 concentration range of 0.5–5 μM.

VX-710 functions in a similar way to the modulators cyclosporin A and PSC 833, influencing the drug accumulation defect by direct interaction with P-g as indicated by photoaffinity labelling experiments. Further evidence of direct high affinity interaction between VX-710 and P-gp is found in the concentration-dependent stimulation of P-gp ATPase activity. The concentrations of VX-710 required to reverse MDR in vitro in a range of cell lines assessed using rhodamine, sestamibi and labelled doxorubicin are similar to cyclosporin A (Germann et al, 1997*a*, 1997*b*). It is unclear to what extent the compound acts as a competitive ligand for binding sites of cytotoxics.

The main attraction of VX-710 in clinical practice is its striking lack of organ toxicity. Other agents active in vitro such as verapamil and high-dose cyclosporin are significantly limited in clinical use, particularly with combination chemotherapy. Both have inherent toxicity and alter chemotherapy pharmacokinetics. Dose-finding studies in adults have demonstrated that the maximum tolerated dose of VX-710 is well in excess of that required to achieve drug levels found to effectively influence drug efflux in vitro. Moreover, preliminary studies in vivo using sestamibi have shown that VX-710 will enhance both hepatic and tumour retention of this surrogate marker for P-gp-related drugs (Peck et al, 1995).

In conclusion, it is apparent that in a range of neuroblastoma cell lines, both VX-710 and PSC 833 are effective sensitizers of MDR-modulated chemotherapy. Although this sensitization is predominantly P-gp-dependent, VX710 did appear to have a broader spectrum of modulator activity. In the two MDR-1-negative lines, which were both MRP-positive, there was some evidence of sensitization using VX-710, which was not apparent with PSC 833 or cyclosporin A. In the MRP control line 2R120, VX-710 was as effective as the other agents in sensitizing to doxorubicin. This is an indication for extension of dose-finding studies in adults to children with refractory or recurrent neuroblastoma or other solid tumours known to be P-gp-positive.

### ACKNOWLEDGEMENTS

This work is supported by the Cancer Research Campaign and the Royal Marsden Hospital Children's Cancer Unit Fund. We thank Vertex Pharmaceutical Incorporated, Novartis Pharmaceuticals and SmithKline Beecham for donating VX-710, PCS 833 and topotecan, respectively.

### REFERENCES

Beck WT, Grogan TM, Willman CL, Cordon Cardo C, Parham DM, Kuttesch JF, Andreeff M, Bates SE, Berard CW, Boyett JM, Brophy NA, Broxterman HJ,

© 1999 Cancer Research Campaign

Chan HS, Dalton WS, Dietel M, Fojo AT, Gascoyne RD, Head D, Houghton PJ and Srivastava DK (1996) Methods to detect P-glycoprotein-associated multidrug resistance in patients' tumors: consensus recommendations. *Cancer Res* **56**: 3010–3020

Beck WT, Handgretinger R, Dopfer R, Klingebiel T, Niethanmmer D and Gekeler V (1995) Expression of MDR1, MRP, topoisomerase Iia/b, and cyclin A in primary or relapsed states of acute lymphoblastic leukaemias. *Br J Haematol* **89**: 356–363

Beketic-Oreskovic L, Duran GE, Chen G, Dumontet G and Sikic BI (1995) Decreased mutation rate for cellular resistance to doxorubicin and suppression of mdr1 gene activation by the cyclosporin PSC 833. *J Natl Cancer Inst* **87**: 1593–1602

Biedler JL and Spengler BA (1976) Metaphase chromosome anomaly: association with drug resistance and cell-specific products. *Science* **191**: 185–187

Biedler JL, Helson L and Spengler BA (1973) Morphology and growth, tumorigenicity and cytogenetics of human neuroblastoma cells in continuous culture. *Cancer Res* **33**: 2652

Boote DJ, Dennis IF, Twentyman PR, Osborne RJ, Laburte C, Hensel S, Smyth JF, Brampton MH and Bleehen NM (1996) Phase I study of etoposide with SDZ PSC 833 as a modulator of multidrug resistance in patients with cancer. *J Clin Oncol* **14**: 610–618

Bordow SB, Haber M, Madafiglio J, Cheung B, Marshall GM and Norris MD (1994) Expression of the multidrug resistance-associated protein (MRP) gene correlates with amplification and overexpression of the N-myc oncogene in childhood neuroblastoma. *Cancer Res* **54**: 5036–5040

Bourhis J, Benard J, Hartmann O, Boccon Gibod L, Lemerle J and Riou G (1989) Correlation of MDR1 gene expression with chemotherapy in neuroblastoma. *J Natl Cancer Inst* **81**: 1401–1405

Cairo MS, Siegel S, Anas N and Sender L (1989) Clinical trial of continuous infusion verapamil, bolus vinblastine, and continuous infusion VP-16 in drug-resistant pediatric tumors. *Cancer Res* **49**: 1063–1066

Chan HS, Haddad G, Thorner PS, DeBoer G, Lin YP, Ondrusek N, Yeger H and Ling V (1991) P-glycoprotein expression as a predictor of the outcome of therapy for neuroblastoma. *N Engl J Med* **325**: 1608–1614

Chan HS, DeBoer G, Haddad G, Gallie BL and Ling V (1995) Multidrug resistance in pediatric malignancies. *Hematol Oncol Clin North Am* **9**: 275–318

Cowie F and Pinkerton CR (1994) Enhanced toxicity of dactinomycin and vincristine by cyclosporine given to reverse multidrug resistance [letter]. *J Clin Oncol* **12**: 1998–1999

Cowie FJ, Pinkerton CR, Phillips M, Dick G, Judson I, McCarthy PT and Flanagan RJ (1995) Continuous-infusion verapamil with etoposide in relapsed or resistant paediatric cancers. *Br J Cancer* **71**: 877–881

Deacon JM (1986) The radiobiology of human neuroblastoma. (A thesis submitted to the University of London for the degree of Doctor of Medicine).

Feller N, Kuiper CM, Lankelma J, Ruhdal JK, Scheper RJ, Pinedo HM and Broxterman HJ (1995) Functional detection of MDR1/P170 and MRP/P190-mediated multidrug resistance in tumour cells by flow cytometry. *Br J Cancer* **72**: 543–549

Futscher BW, Blake LL, Gerlach JG, Grogan TM and Dalton WS (1993) Quantitative polymerase chain reaction analysis of mdr1 mRNA in multiple myeloma cell lines and clinical specimens. *Analyt Biochem* **213**: 421

Germann UA, Shlyakher D, Mason VS, Zelle RE. Duffy JP, Galullo V, Armistead DM, Saunders JO, Boger J and Harding MW (1997*a*) Cellular and biochemical characterization of VX-710 as a chemosensitizer: reversal of P-glycoprotein-mediated multidrug resistance in vitro. *Anti-Cancer Drugs* **8**: 125–140

Germann UA, Ford PJ, Shlyakher D, Mason VS and Harding MW (1997*b*) Chemosensitization and drug accummulation effects of VX-710, verapamil, cyclosporin A, MS-209 and GF120918 in multidrug resistant HL60/ADR cells expressing the multidrug resistance-associated protein MRP. *Anti-Cancer Drugs* **8**: 141–155

Goodwin CJ, Holt SJ, Downes S and Marshall NJ (1995) Microculture tetrazolium assays: a comparison between two new tetrazolium salts, XTT and MTS. *J Immunol Methods* **179**: 95–103

Helson C, Zahn Z and Helson L (1994) Reversion of P-glycoprotein mediated multi-drug resistance to vincristine and adriamycin by PSC 833, a cyclosporine derivative in human neuroblastoma cell lines. *Int J Oncol* **5**: 1037–1042

Hills CA, Kelland LR, Abel G, Siracky J, Wilson AP and Harrap KR (1989) Biological properties of ten human ovarian carcinoma cell lines: calibration in vitro against four platinum complexes. *Br J Cancer* **59**: 527–534

Inui A (1974) Catecholamine metabolism in a continuous human neuroblastoma cell line (NB-1). Establishment of a continuous cell line (NB-1) and comparative study on catecholamine metabolism in neuroblastoma in vivo and in vitro. *Acta Pediatr Jpn* **78**: 977–989

Lum BL, Fisher GA, Brophy NA, Yahanda AM, Adler KM, Kaubisch S, Halsey J and Sikic BI (1993) Clinical trials of modulation of multidrug resistance. Pharmacokinetic and pharmacodynamic considerations. *Cancer* **72**: 3502–3514

Norris MD, Bordow SB, Marshall GM, Haber PS, Cohn SL and Haber M (1996) Expression of the gene for multidrug-resistance-associated protein and outcome in patients with neuroblastoma. *New England Journal of Medicine* **334**: 231–238

Peck R, Marshall JL, Ziessman HA, et al. (1995) Preliminary findings from a phase I/II trial of doxorubicin (DOX) and VX-710. [Abstract] AACR, Special Conference in Cancer Research: Novel Strategies Against Resistant Cancers

Pike BL and Robinson WA (1970) Human bone marrow colony growth in agar-gel. *J Cell Pysiol* **76**: 77–84

Rowinsky EK, Smith I, Wang YM et al (1998) Phase I and pharmacokinetic study of paclitaxel in combination with Biricodar, a Novel Agent that reverses multidrug resistance conferred by overexpression of both MDR1 and MRP. *J Clin Oncol* **16**: 2964–2976

Stein U, Walther W, Laurencot CM, Scheffer GL, Scheper RL and Shoemaker RH (1997) Tumor necrosis factor-α and expression of the multidrug resistance associated genes LRP and MRP. *J Natl Cancer Inst* **89**: 807–813

Tumilowicz JJ, Nichols WW, Cholon JJ and Greene AE (1970) Definition of a continuous human cell line derived from neuroblastoma. *Cancer Res* **30**: 2110–2118

*© 1999 Cancer Research Campaign*
